# Growth and Structure of ZnO Nanorods on a Sub-Micrometer Glass Pipette and Their Application as Intracellular Potentiometric Selective Ion Sensors 

**DOI:** 10.3390/ma3094657

**Published:** 2010-09-09

**Authors:** Muhammad H. Asif, Omer Nur, Magnus Willander, Peter Strålfors, Cecilia Brännmark, Fredrik Elinder, Ulrika H. Englund, Jun Lu, Lars Hultman

**Affiliations:** 1 Department of Science and Technology, Campus Norrköping, Linköping University, SE-60174 Norrköping Sweden; E-Mails: omeno@itn.liu.se (O.N.); magwi@itn.liu.se (M.W.); 2 Department of Clinical and Experimental Medicine, Division of Cell Biology, Linköping University, SE-58185 Linköping, Sweden; E-Mails: peter.stralfors@liu.se (P.S.); cecilia.brannmark@gmail.com (C.B.); fredrik.elinder@liu.se (F.E.); ulrika.englund@liu.se (U.H.E.); 3 Department of Physics (IFM), Linkoping University, SE-581 83 Linköping, Sweden; E-Mails: junlu@ifm.liu.se (J.L.); larhu@ifm.liu.se (L.H.)

**Keywords:** ZnO nanorods, HRTEM, functionalization, membrane, intracellular sensor

## Abstract

This paper presents the growth and structure of ZnO nanorods on a sub-micrometer glass pipette and their application as an intracellular selective ion sensor. Highly oriented, vertical and aligned ZnO nanorods were grown on the tip of a borosilicate glass capillary (0.7 µm in diameter) by the low temperature aqueous chemical growth (ACG) technique. The relatively large surface-to-volume ratio of ZnO nanorods makes them attractive for electrochemical sensing. Transmission electron microscopy studies show that ZnO nanorods are single crystals and grow along the crystal’s c-axis. The ZnO nanorods were functionalized with a polymeric membrane for selective intracellular measurements of Na^+^. The membrane-coated ZnO nanorods exhibited a Na^+^-dependent electrochemical potential difference *versus* an Ag/AgCl reference micro-electrode within a wide concentration range from 0.5 mM to 100 mM. The fabrication of functionalized ZnO nanorods paves the way to sense a wide range of biochemical species at the intracellular level.

## 1. Introduction

ZnO nanostructures receive growing attention for electronics, optics and photonics. Various ZnO nanostructures such as nanowires and nanorods, synthesized by diverse methods, show valuable properties [1,2]. Nanodevice functionality has been demonstrated with these one-dimensional (1D) semiconducting nanostructure materials in the form of electric field-effect switching [3], single electron transistors [4], biological and chemical sensing [5], and luminescence [6]. 

The structure of ZnO can be described by alternating planes composed of tetrahedral coordinated O^2-^ and Zn^2+^ ions, stacked alternately along the c-axis [7] with ionicity of around 60%. ZnO is a piezoelectric, bio-safe and biocompatible material. It is a polar semiconductor with two crystallographic planes with opposite polarity and different surface relaxation energies. This leads to a higher growth rate along the c-axis. The oppositely charged ions produce positively charged Zn-(0001) and negatively charged O-(000-1) polar surfaces, resulting in a normal dipole moment and spontaneous polarization along the c-axis. The crystal structures formed by ZnO are wurtzite, zinc blende, and rocksalt. Due to the small dimensions combined with drastically increased contact surface and strong binding with biological and chemical reagents, ZnO nanowires and nanorods have the potential for important applications in biological and biochemical research. The diameter of these nanostructures is usually comparable to the size of the biological and chemical species being sensed, which promise excellent primary transducers for producing electrical signals. Especially when the diameter of ZnO nanostructures reaches a few nanometers, it is expected that their properties could be affected by the structure of the side surface. 

For applications, it is important to determine the surface structure of 1D ZnO nanostructures. Transmission electron microscopy (TEM) is a powerful tool for characterizing such nanostructures. It is important not only in determining the crystal and surface structure, but also the chemical composition [8]. One of the important potential applications of ZnO nanorods is its use in chemical sensors. Due to large surface-to-volume ratios, ZnO nanorods have been demonstrated as a candidate for highly sensitive, nanosized chemical sensors [9,10]. Recently, the electrical and chemical sensing properties of ZnO nanorods have been extensively investigated. Literature surveys reveal that ZnO nanorods are n-type semiconductors and their electrical transport depends on the adsorption/desorption nature of contacting chemical species [11,12,13,14,15,16]. 

Sodium ions, Na^+^, are abundant in the extracellular space and have an important role in the excitability of nerve and muscle cells, and in water and salt homeostasis in biological systems [17,18,19]. While the intracellular concentration of Na^+^ is typically low in such environments, the role of intracellular Na^+^ has not been fully understood. However, during the last decade several studies have revealed that Na^+^ may act as a second messenger in different cell types. Na^+^ regulates different transporters and receptors in renal cells and neurons, and it regulates the activation of both Na^+^ and K^+^ channels, thereby regulating the reabsorption of salt and water in the kidneys and neuronal excitability [20,21,22,23,24,25]. Na^+^ channels and the intracellular concentration of Na^+^ are important in the early stages of apoptotic processes in cell lines, and Na^+^ may be involved in intracellular signaling during apoptosis [26,27,28,29,30].

Here, we describe the application of ZnO nanorods inside cells. The use of ZnO nanorods for intracellular detection of biological analytes, metallic ions, and clusters has been developed by our group. We have performed preliminary intracellular detection of most of the basic metallic ions, and glucose in oocytes and adipocyte cells [31,32]. The objective of this article is to characterize ZnO nanorods and present their application as an intracellular potentiometric selective ion sensor. Intracellular determination of Na^+^ is of great interest and ZnO nanorod technology has the potential for such measurements. We demonstrate a ZnO nanorod-based sensor suitable for intracellular selective Na^+^ detection as well the optimization of its electrochemical properties. This sensor is based on ZnO nanorods grown on a Borosilicate glass capillary (sterile Femtotip® II with tip inner diameter of 0.5 µm and a tip outer diameter of 0.7 µm, and length of 49 mm) that is capable of penetrating a cell membrane. 

## 2. Results and Discussion

**Figure 1:**
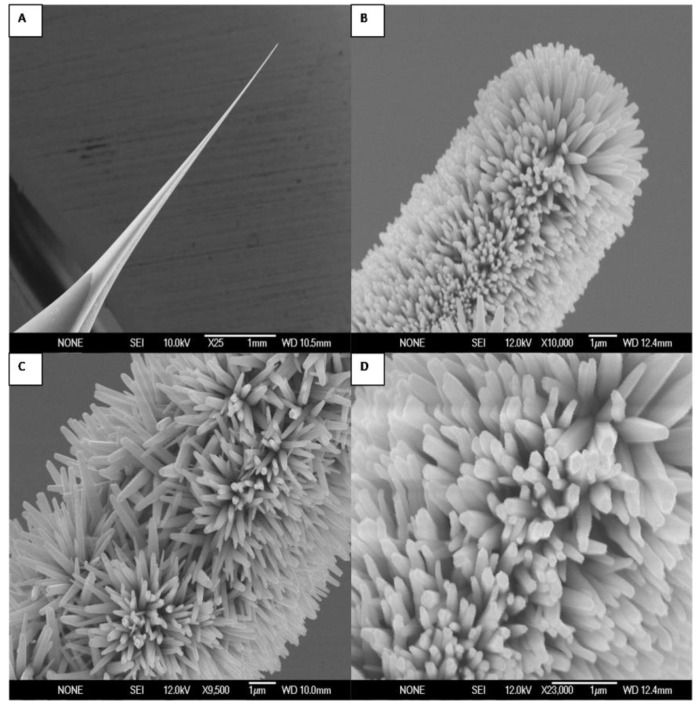
Field emission scanning electron microscope images at different magnifications of the Ag-coated glass capillary without (**A**) and with (**B**–**D**) grown ZnO nanorods using low temperature aqueous chemical solution growth.

The morphology of the as-grown high-density and aligned ZnO nanorods was investigated by field emission scanning electron microscopy (FESEM). Figure 1 shows typical FESEM images, at different magnifications, showing that the ZnO nanorods grown on the glass tip substrate have a rod-like shape with a hexagonal cross section and are primarily aligned along the surface perpendicular direction. Previous studies have shown that ZnO nanorods have the wurtzite structure (hexagonal) and grow along the c-axis direction [38].

**Figure 2:**
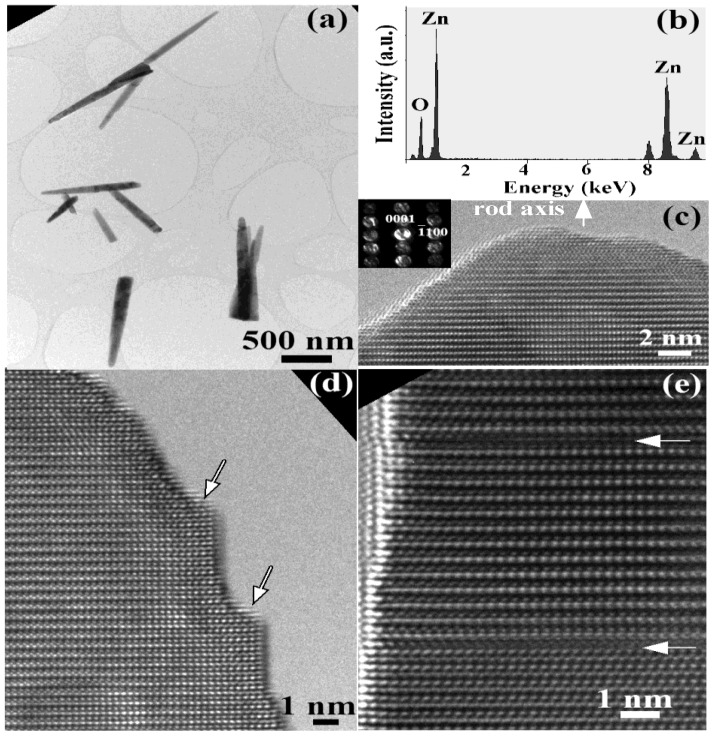
(**A**, **D**, **E**) TEM images; (**B**) EDX analysis; and (**C**) SAED pattern from typical ZnO nanorods.

The typical diameter of the as-grown nanorods is in the range of 50 to 70 nm and the length is in the range of about 1 µm (figure 2(a)). Chemical composition microanalysis by energy dispersive X-Ray (EDX) demonstrates that the rods consist only of O and Zn without other metal impurities as catalysts (see figure2 (b)). The additional peak at 8 keV is Cu Kα from the copper grid. The high-resolution transmission electron microscopy (HRTEM) image and selected area electron diffraction (SAED) pattern are shown in figure 2 (c) with insert revealing that the nanorod long axis is along the [0001] direction. The rods contain some defects such as surface steps and stacking faults shown by arrows in figure 2 (d, e).

The selectivity was tested with 1 mM calcium (Ca^2+^), magnesium (Mg^2+^), or potassium (K^+^) added to a 1-mM NaCl solution. There was no observable effect on the Na^+^ response. However, introduction of K^+^ at high concentrations revealed that the sensor signal was clearly affected by K^+^ through slower response and lower stability. This implies that the sensor possesses good selectivity through the use of membrane containing ionophore at physiological concentrations. 

**Figure 3:**
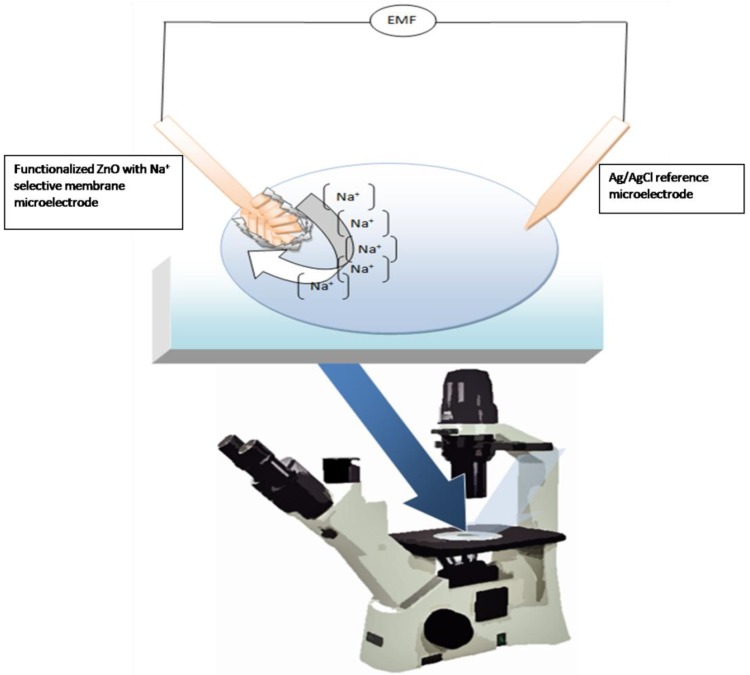
Schematic diagram illustrating the setup for the measurement of the intracellular Na^+^ concentration.

A simple potentiometric technique for determining the Na^+^ concentration in biological compartments was then developed and the experimental setup for intracellular measurements is presented in Figure 3. 

**Figure 4:**
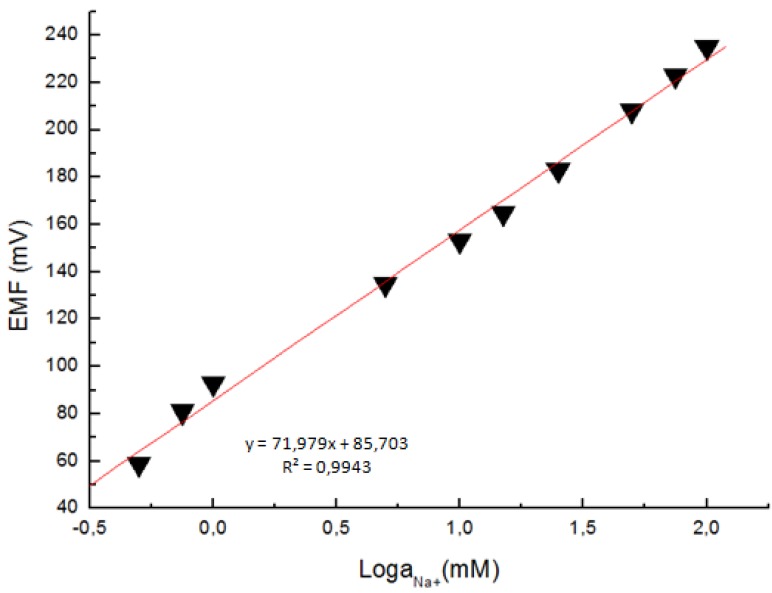
A calibration curve showing the electrochemical potential difference between the Na^+^-selective ZnO nanorod and the Ag/AgCl reference microelectrodes *versus* the Na^+^ concentration.

Microelectrodes with a tip diameter about 1 µm were used for the potentiometric measurement of the intracellular Na^+^ concentration. The electrochemical cell voltage (electromotive force) is changed when the composition of the test electrolyte is changed. These changes can be related to the concentration of ions in the test electrolyte via a calibration procedure shown in Figure 4. The actual electrochemical potential cell can be described by the diagram below:

[Ag | ZnO | buffer || Cl^−^ |AgCl | Ag] (1)

The response of the electrochemical potential difference of the ZnO nanorods to the changes in buffer electrolyte Na^+^ was measured with a range from 0.5 mM to 100 mM. This demonstrates that the Na^+^ dependence is linear and has sensitivity of to 72 mV/decade at around 23 °C (Figure 4). This linear dependence implies that such sensor configuration can provide a large dynamical range.

We test these nanostructures for natural signal transduction elements when dealing with the detection of biological analytes, metallic ions, and clusters. Here, we note that ZnO is also a semiconducting material that is bio-safe and biocompatible and possesses excellent signal transmission properties.

The functionalized microelectrodes were then used to measure the free concentration of intracellular Na^+^ in a single human adipocyte. The Na^+^ selective microelectrode was, mounted on a micromanipulator, and moved into a position at the same level as the cells. The ZnO-based and the reference microelectrodes were gently pushed through the cell membrane and into the cell (Figure 3). Once the ZnO nanorod and the Ag/AgCl reference microelectrodes were inside the cell, that is isolated from the surrounding buffer solution, an electrochemical potential difference signal was detected. The intracellular Na^+^ concentration was 11.5 mM, corresponding closely to the earlier reported intracellular concentrations reported in the literature [39]. In a similar experiment we used the nanosensor to measure the intracellular Na^+^ concentration in single frog oocytes using the same setup as for the adipocytes, the intracellular Na^+^ concentration in frog oocytes was 8 mM, which is close to what has been reported before in [40]. 

Studying the solubility of ZnO nanorods in biofluids has important implications for its applications in biomedical science. Firstly, ZnO has the potential to be used for biosensors, where it requires a reasonable time to function in biological systems and perform a device function. Secondly, if the ZnO nanorods are left in the body or in a blood vessel, they will be dissolved by the biofluid into non-toxic ions that may be absorbed by the body and become part of the nutrition, as Zn ions are needed for the human body [41]. The ZnO nanorod based microelectrodes described here are designed for intracellular use. A first series of intracellular measurements has been successfully conducted [31,32]. 

The viability of the cells depends on the size of the ZnO nanorods, time and heating effect due to the microscope. We can improve the viability of cells by controlling these parameters.

## 3. Experimental Section

### 3.1. Growth and characterization of ZnO nanostructures

Well-controlled and aligned ZnO nanostructures were prepared by aqueous chemical growth (ACG), which is a common and cost-effective low-temperature technique. The growth procedure is as follows: the ZnO nanorods were grown on Ag coated tips of borosilicate glass capillaries in a solution of zinc nitrate hexahydrate [Zn(NO_3_)_2_·6H_2_O, 99.9% purity] and hexamethylenetetramine (C_6_H_12_N_4_, 99.9% purity). The concentrations of both were fixed at 0.025 M. All the aqueous solutions were prepared in distilled water and we restrict the results to glass tip substrate. The glass capillaries substrates were immersed into the solution and tilted against the wall of the beaker. After that, the beaker was put into the oven at around 93 °C for different times to get aligned ZnO nanostructure. Then the substrate was removed from the solution and cleaned with de-ionized water. The as-grown ZnO nanorods on glass tip have been studied by FESEM at different magnifications. The ZnO nanorods were also characterized with HRTEM using a Tecnai G2 UT instrument operated at 200 kV with 0.19 nm point resolution. The TEM specimen was made by scraping the nanorods onto a copper grid with carbon film.

### 3.2. Immobilization of membrane and electrochemical measurements 

The composition of the polyvinyl chloride (PVC) membrane-based Na^+^ electrode is given as 1% Na^+^ ionophore ETH 227, 0.5% Sodium tetraphenylborate, 75% *o*-Nitrophenyl octyl ether, and 23.5% PVC. The solution of plastic membrane components is prepared in 5 mL tetrahydrofuran (THF). The ZnO nanorod layer on the silver-coated microelectrode was coated with ionophore-containing liquid polymeric membrane by a manual procedure. This ionophore exhibits the best result for potentiometric sensing of Na^+^ [33]. All chemicals were purchased from Sigma-Aldrich. The ZnO-coated microelectrode was dipped twice into the prepared solution. After each dip the electrodes were allowed to dry at room temperature. Finally to condition the microelectrode for selectivity of Na^+^, it was dipped into a 10 mM NaCl_2_ solution [34].

In a complete potentiometric cell, the Na^+^-selective microelectrode is used in conjunction with a reference microelectrode. The electrochemical potential between the Na^+^-selective and the Ag/AgCl reference microelectrodes was measured with Metrohm pH meter model 827.

### 3.3. Cellular preparations 

Two types of cells were used for intracellular Na^+^ measurements; human adipocytes and frog oocytes. Primary human adipocytes (fat cells) were isolated by collagenase digestion of pieces of subcutaneous adipose tissue [35] obtained during elective surgery at the university hospital in Linköping, Sweden. Cells were incubated overnight before use as described in [36]. For the experiments, cells were transferred to a modified Krebs-Ringer solution buffered with 20 mM Hepes, pH 7.4, as detailed in [36]. A glass slide (5 cm length, 4 cm width, and 0.17 mm thickness) with sparsely distributed fat cells was placed on the pre-warmed microscope stage set at 37 °C. The Na^+^-selective and the reference microelectrodes, mounted on a micromanipulator, were gently manipulated a short way into the cell by using hydraulic fine adjustments, through the cell membrane.

Oocytes were isolated from ovarian lobes cut off through a small abdominal incision from female *Xenopus laevis* frogs anesthetized in a bath with tricaine (procedure approved by the local Animal Care and Use Committee at Linköping University). Stage V and VI oocytes (approximately 1 mm in diameter) without spots and with clear delimitation between the animal and vegetal pole were selected. All details regarding preparation of oocytes and the solutions used are described in [37]. 

## 4. Conclusions

Highly oriented ZnO nanorods have been grown on the tip of a borosilicate glass capillary (0.7 µm in diameter) by a low-temperature ACG technique. The ZnO nanorods were characterized by HRTEM, which shows that the nanorods are single crystalline and structurally uniform with a diameter ranging from 50 to 70 nm and a length of up to 1 µm. The nanorods grow along the [0001] crystallographic direction. The biocompatibility and bio-safety of ZnO nanorods were utilized to develop an intracellular potentiometric selective Na^+^ sensor. Successful invasive intracellular measurement using such ZnO nanorods is demonstrated. The ZnO nanorods were functionalized by covering them with the Na^+^-selective membrane. The potential difference between the Na^+^-selective and the reference microelectrodes was found to be linear over a wide concentration range of Na^+^ (0.5 mM to 100 mM). Human adipocytes and frog oocytes were used for intracellular measurements. The measured intracellular Na^+^ concentrations in single human adipocytes and frog oocytes were consistent with values found in the literature. The results from the electrochemical sensors indicate the potential and relevance of using ZnO nanorods in biological and biochemical environment monitoring.

## References

[1] Heo Y.W., Norton D.P., Tien L.C., Kwon Y., Kang B.S., Ren F., Pearton S.J., LaRoche J.R. (2004). ZnO nanowire growth and devices. Mater. Sci. Eng..

[2] Fan Z., Lu J.G. (2005). Zinc oxide nanostructures: Synthesis and properties. J. Nanosci. Nanotechnol..

[3] Kim G.T., Muster J., Krstic V., Park J.G., Park Y.W., Roth S., Burghard M. (2000). Field-effect transistor made of individual V_2_O_5_ nanofibers. Appl. Phys. Lett..

[4] Stone N.J., Ahmed H. (1998). Silicon single electron memory cell. Appl. Phys. Lett..

[5] Cui Y., Wei Q., Park H., Lieber C.M. (2001). Nanowire nanosensors for highly sensitive and selective detection of biological and chemical species. Science.

[6] Huang M.H., Mao S., Feick H., Yan H., Wu Y., Kind H., Weber E., Russo R., Yang P. (2001). Room-temperature ultraviolet nanowire nanolasers. Science.

[7] Wang Z.L., Kong X.Y., Ding Y., Gao P., Hughes W.L., Yang R., Zhang Y. (2004). Semiconducting and piezoelectric oxide nanostructures induced by polar surface. Adv. Funct. Mater..

[8] Ding Y., Wang Z.L. (2004). Structure analysis of nanowires and nanobelts by transmission electron microscopy. J. Phys.Chem. B.

[9] Tien L.C., Sadik P.W., Norton D.P., Voss L.F., Pearton S.J., Wang H.T., Kang B.S., Ren F., Jun J., Lin J. (2005). Hydrogen sensing at room temperature with Pt-coated ZnO thin films and nanorods. Appl. Phys. Lett..

[10] Hsueh T.J., Chang S.J., Hsu C.L., Lin Y.R., Chen I.C. (2007). Highly sensitive ZnO nanowire ethanol sensor with Pd adsorption. Appl. Phys. Lett..

[11] Li Q.H., Gao T., Wang Y.G., Wang T.H. (2005). Adsorption and desorption of oxygen probed from ZnO nanowire films by photocurrent measurements. Appl. Phys. Lett..

[12] Hung X.J., Choi Y.K. (2007). Chemical sensors based on nanostructured materials. Sensor. Actuator. B-Chem..

[13] Li C.C., Du Z.F., Li L.M., Yu H.C., Wan Q., Wang T.H. (2007). Surface-depletion controlled gas sensing of ZnO nanorods grown at room temperature. Appl. Phys. Lett..

[14] Ghosh R., Dutta M., Basak D. (2007). Self-seeded growth and ultraviolet photoresponse properties of ZnO nanowire arrays. Appl. Phys. Lett..

[15] Qiu Y.F, Yang S.H. (2007). ZnO nanotetrapods: Controlled vapor-phase synthesis and application for humidity sensing. Adv. Funct. Mater..

[16] Park J.Y., Song D.E., Kim S.S. (2008). An approach to fabricating chemical sensors based on ZnO nanorod arrays. Nanotechnology.

[17] Hille B. (2001). Ion Channel of Excitable Membranes.

[18] McDonough A.A. (2010). Mechanisms of proximal tubule sodium transport regulation that link extracellular fluid volume and blood pressure. Am. J. Physiol. Regul. Integr. Comp. Physiol..

[19] Hodgkin A.L., Katz B. (1949). The effect of sodium ions on the electrical activity of the giant axon of the squid. J. Physiol. (Lond.).

[20] Knight K.K., Wentzlaff D.M., Snyder P.M. (2008). Intracellular sodium regulates proteolytic activation of the epithelial sodium channel. J. Biol. Chem..

[21] Ho I.H., Murrell-Lagnado R.D. (1999). Molecular mechanism for sodium-dependent activation of G protein-gated K^+^ channels. J. Physiol..

[22] Yu X.M., Salter M.W. (1998). Gain control of NMDA-receptor currents by intracellular sodium. Nature.

[23] Efendiev R., Bertorello A.M., Zandomeni R., Cinelli A.R., Pedemonte C.H. (2002). Agonist-dependent regulation of renal Na^+^, K^+^-ATPase activity is modulated by intracellular sodium concentration. J. Biol. Chem..

[24] Budelli G., Hage T.A., Wei A., Rojas P., Jong Y.J., O’Malley K., Salkoff L. (2009). Na^+^-activated K^+^ channels express a large delayed outward current in neurons during normal physiology. Nat. Neurosci..

[25] Kameyama M., Kakei M., Sato R., Shibasaki T., Matsuda H., Irisawa H. (1984). Intracellular Na^+^ activates a K^+^ channel in mammalian cardiac cells. Nature.

[26] Akanda N., Tofighi R., Brask J., Tamm C., Elinder F., Ceccatelli S. (2008). Voltage-dependent anion channels (VDAC) in the plasma membrane play a critical role in apoptosis in differentiated hippocampal neurons but not in neural stem cells. Cell Cycle.

[27] Thompson G.J., Langlais C., Cain K., Conley E.C., Cohen G.M. (2001). Elevated extracellular [K^+^] inhibits death-receptor- and chemical-mediated apoptosis prior to caspase activation and cytochrome c release. Biochem. J..

[28] Banasiak K.J., Burenkova O., Haddad G.G. (2004). Activation of voltage-sensitive sodium channels during oxygen deprivation leads to apoptotic neuronal death. Neuroscience.

[29] Bortner C.D., Cidlowski J.A. (2003). Uncoupling cell shrinkage from apoptosis reveals that Na^+^ influx is required for volume loss during programmed cell death. J. Biol. Chem..

[30] Bortner C.D., Gomez-Angelats M., Cidlowski J.A. (2001). Plasma membrane depolarization without repolarization is an early molecular event in anti-Fas-induced apoptosis. J. Biol. Chem..

[31] Asif M.H., Fulati A., Nur O., Willander M., Brännmark C., Strålfors P., Börjesson S.I., Elinder F. (2009). Functionalized zinc oxide nanorod with ionophore-membrane coating as an intracellular Ca^2+^ selective sensor. Appl. Phys. Lett..

[32] Asif M.H., Ali S.U., Nur O., Willander M., Brännmark C., Strålfors P., Englund U.H., Elinder F., Danielsson B. (2010). Functionalised ZnO-nanorod-based selective electrochemical sensor for intracellular glucose. Biosens. Bioelectron..

[33] Michalska A., Hulanicki A., Lewenstam A. (1997). All-Solid-State Potentiometric Sensors for Potassium and Sodium Based on Poly(pyrrole) Solid Contact. Microchem. J..

[34] Asif M.H., Nur O., Willander M., Yakovleva M., Danielsson B. (2008). Studies on calcium ion selectivity of ZnO nanowire sensors using ionophore membrane coatings. Res. Lett. Nanotechnol..

[35] Strålfors P., Honnor R.C. (1989). Insulin-induced dephosphorylation of hormone-sensitive lipase Correlation with lipolysis and CAMP-dependent protein kinase activity. Eur. J. Biochem..

[36] Danielsson A., Öst A., Lystedt E., Kjolhede P., Gustavsson J., Nyström F.H., Strålfors P. (2005). Insulin resistance in human adipocytes occurs downstream of IRS1 after surgical cell isolation but at the level of phosphorylation of IRS1 in type 2 diabetes. FEBS J..

[37] Börjesson S.I., Parkkari T., Hammarström S., Elinder F. (2010). Electrostatic tuning of cellular excitability. Biophys. J..

[38] Wang Z.L. (2009). ZnO nanowire and nanobelt platform for nanotechnology. Mater. Sci. Eng..

[39] Resh M.D., Nemenoff R.A., Guidotti G. (1980). Insulin stimulation of (Na^+^, K^+^)- adenosine triphosphatase-dependent 86Rb^+^ uptake in rat adipocytes. J. Biol. Chem..

[40] Dascal N. (1987). The use of Xenopus oocytes for the study of ion channels. CRC Crit. Rev. Biochem..

[41] Zhou B.J., Xu N., Wang Z.L. (2006). Dissolving behavior and stability of ZnO wires in biofluids: A study on biodegradability and biocompatibility of ZnO nanostructures. Adv. Mater..

